# Tallness and overweight during childhood have opposing effects on breast cancer risk

**DOI:** 10.1054/bjoc.2001.2109

**Published:** 2001-11

**Authors:** L Hilakivi-Clarke, T Forsén, J G Eriksson, R Luoto, J Tuomilehto, C Osmond, D J P Barker

**Affiliations:** Lombardi Cancer Center, Georgetown University, Washington, DC, 20007, USA; Department of Epidemiology and Health Promotion, Diabetes and Genetic Epidemiology Unit, National Public Institute, Helsinki, FIN-00300, Finland; Department of Health and Disability, National Public Institute, Helsinki, FIN-00300, Finland; MRC Environmental Epidemiology Unit, University of Southampton, Southampton General Hospital, Southampton, SO16 6YD, United Kingdom

**Keywords:** breast cancer, body mass, height, childhood

## Abstract

Using birth and school health records we studied how weight and height during childhood affect breast cancer risk among 3447 women born during 1924–33 at the University Hospital of Helsinki, Finland. Through linkages with the National Hospital Discharge Registry and the Cause of Death Registry we identified177 women who during 1971–1995 had been admitted to hospital with breast cancer, of whom 49 had died from the disease. Of these, 135 (76%) were aged 50 years or more at the time of diagnosis, and therefore likely to have been post-menopausal. Hazard ratios for breast cancer rose with increasing weight and length at birth, though neither trend was statistically significant. At each age, from 7 to 15 years, the girls who later developed breast cancer were on average taller and had lower body mass than the other girls. Unadjusted hazard ratios rose across the range of height (*P* = 0.01 at age 7 years) and fell across the range of body mass index (*P* = 0.009 at age 7 years). In a simultaneous analysis the hazard ratio for breast cancer was 1.27 (95% CI 0.97–1.78, *P* = 0.08) for every kilogram increase in birth weight and 1.21 (95% CI 1.06–1.38, *P* = 0.004) for every kg/m^2^ decrease in body mass index at 7. Our findings indicate that tallness in childhood is associated with increased risk of developing breast cancer. One possible explanation is persisting high plasma concentrations of insulin-like growth factors in talll women. In contrast, we found that being overweight in childhood reduces breast cancer risk. The increased adipose tissue-derived oestrogen levels in overweight children could induce early breast differentiation and eliminate some targets for malignant transformation. © 2001 Cancer Research Campaign http://www.bjcancer.com


*British Journal of Cancer* (2001) **85**(11), 1680–1684
© 2001 Cancer Research Campaign
doi: 10.1054/ bjoc.2001.2109, available online at http://www.idealibrary.com on IDEAL
http://www.bjcancer.com

### Tallness and overweight during childhood have opposing effects on breast cancer risk

**L Hilakivi-Clarke[1], T Forsén[2], JG Eriksson[2], R Luoto[3], J Tuomilehto[2], C Osmond[4] and DJP Barker[4]**

[1]Lombardi Cancer Center, Georgetown University, Washington, DC 20007, USA; [2]Department of Epidemiology and Health Promotion, Diabetes and Genetic Epidemiology Unit, National Public Institute, FIN-00300 Helsinki, Finland; [3]Department of Health and Disability, National Public Institute, FIN-00300 Helsinki, Finland and [4]MRC Environmental Epidemiology Unit, University of Southampton, Southampton General Hospital, Southampton, SO16 6YD, United Kingdom

**Summary** Using birth and school health records we studied how weight and height during childhood affect breast cancer risk among 3447 women born during 1924–33 at the University Hospital of Helsinki, Finland. Through linkages with the National Hospital Discharge Registry and the Cause of Death Registry we identified177 women who during 1971–1995 had been admitted to hospital with breast cancer, of whom 49 had died from the disease. Of these, 135 (76%) were aged 50 years or more at the time of diagnosis, and therefore likely to have been post-menopausal. Hazard ratios for breast cancer rose with increasing weight and length at birth, though neither trend was statistically significant. At each age, from 7 to 15 years, the girls who later developed breast cancer were on average taller and had lower body mass than the other girls. Unadjusted hazard ratios rose across the range of height ($P = 0.01$ at age 7 years) and fell across the range of body mass index ($P = 0.009$ at age 7 years). In a simultaneous analysis the hazard ratio for breast cancer was 1.27 (95% CI 0.97–1.78, $P = 0.08$) for every kilogram increase in birth weight and 1.21 (95% CI 1.06–1.38, $P = 0.004$) for every kg/m$^2$ decrease in body mass index at 7. Our findings indicate that tallness in childhood is associated with increased risk of developing breast cancer. One possible explanation is persisting high plasma concentrations of insulin-like growth factors in talll women. In contrast, we found that being overweight in childhood reduces breast cancer risk. The increased adipose tissue-derived oestrogen levels in overweight children could induce early breast differentiation and eliminate some targets for malignant transformation. © 2001 Cancer Research Campaign http://www.bjcancer.com

**Keywords**: breast cancer; body mass; height; childhood

It has been hypothesized that accelerated growth during fetal life or an enhanced oestrogenic environment in utero increases subsequent breast cancer risk (Trichopoulos, 1990). This hypothesis is supported by limited indirect epidemiological evidence, including studies showing that high birth weight and being a dizygotic twin, both of which are associated with elevated pregnancy oestrogen levels (Gerhard et al, 1987; Johnson et al, 1994), increase the risk of developing breast cancer (Braun et al, 1995; Cerhan et al, 2000; Michels et al, 1996; Sanderson et al, 1996). Animal studies show that maternal exposure to oestradiol during pregnancy or a high fat diet that elevates pregnancy oestradiol levels, increase carcinogen-induced mammary tumorigenesis among female offspring (Hilakivi-Clarke et al, 1997).

Relatively little is known about growth during childhood and breast cancer. Tall or obese women are at an increased risk of developing postmenopausal breast cancer (Cold et al, 1996; Vatten and Kvinnsland, 1990; Yong et al, 1989; Ziegler, 1997). Thin women are more likely than obese women to develop pre-menopausal breast cancer (Cleary and Maihle, 1997; Huang et al, 1997; Potischman et al, 1996; Trentham-Dietz et al, 1997). Three recent studies suggest that high childhood or adolescent body mass index may protect against breast cancer risk (Berkey et al, 1999; Le Marchand et al, 1988; Magnusson et al, 1998). Many investigators, however, believe the opposite and propose that childhood obesity increases the risk (Colditz and Frazier, 1995; deWaard and Trichopoulos, 1988; Hunter and Willett, 1996). This belief is based on the association between childhood obesity and early onset of puberty (Frisch and McArthur, 1974), since early puberty is consistently related to increased risk of breast cancer (Hulka and Stark, 1995).

We have studied a cohort of 3447 Finnish women whose size at birth and childhood growth were recorded, to examine whether height and body mass index during childhood affected breast cancer risk. We also examined whether childhood growth modified the effects of birth size on risk.

### METHODS

The study cohort comprised women who were born at the Helsinki University Central Hospital during 1924–1933, who went to school in the city of Helsinki, and were still residents in Finland as of 1971. The 11-year birth period chosen gives a cohort of women old enough to have experienced pre-and post-menopausal breast cancer. Details of the birth records kept by the Helsinki University Central Hospital have been described previously (Forsen et al, 1997). School health records for all children in Helsinki are stored in the city archive. Beginning in 1971, all residents of Finland have been assigned a unique personal identification number. From the birth and school health records and identification numbers, we identified 3688 women for use in our study (Forsen et al, 1999). Of these women, 241 subsequently emigrated and the date of emigration was not always recorded. We therefore excluded them from the study to leave 3447 women.

*Received 6 April 2001*
*Revised 23 July 2001*
*Accepted 1 August 2001*

*Correspondence to:* L Hilakivi-Clarke

Recorded data on the newborn babies included birthweight, length, head circumference and placental weight. Data on their mothers included age, parity, height and date of the last menstrual period, together with body weight, measured on admission in labour. The school records included an average of 10 (± SD 4) measurements of height and weight between the ages of 6 and 16 years, recorded during periodic medical examinations. Age at menarche was not recorded. Further, no information was available of the factors after menarche that are known to be related to breast cancer risk, i.e., age at menopause, adult body weight, parity, or age at first pregnancy.

By using the personal identification number, we identified all hospital admissions and deaths among the women during 1971–1995. All hospital admissions in Finland are recorded in the national hospital discharge register. All deaths are recorded in the national mortality register. Causes of hospital admissions or death were recorded according to ICD-8 (international classification of diseases, 8th revision) until 1986; thereafter ICD-9 was used until 1995. The first three digits from the cause of admission or death were used to identify breast cancer cases in the cohort (174 in ICD-8 and ICD-9).

### Statistical methods

We examined the trends in hazard ratios with maternal, neonatal and childhood measurements. Tests for trend were based on Cox's proportional hazards model. Each measurement of height, weight and body mass index for each girl was converted to a Z score (Royston, 1991). We then interpolated between successive Z scores with a piecewise linear function and so obtained a Z score at each birthday from age 7 to 15 years. The Z scores were back transformed to obtain the corresponding height, weight and body mass index at these ages, as previously described (Forsen et al, 1999). Growth velocity was measured as the change in Z scores between ages 7 and 15 years.

### RESULTS

Maternal, neonatal and childhood characteristics of the 3447 women are shown in Table 1. We found that 177 of these women were admitted to hospital with breast cancer, of whom 49 died from the disease. The annual death rate from breast cancer at ages 45–64 years was 6.1 per 10 000. In 1971, when the first breast cancer cases were ascertained, the women's ages ranged from 38 to 47 years. Although most women would have been pre-menopausal at that time, 135 (76%) of the 177 women who developed breast cancer during the follow-up, were aged 50 years or more at the time of diagnosis and were therefore likely to have been post-menopausal. In the analyses which follow we found no differences in the association with breast cancer first diagnosed below and over the age of 50 years.

### Size at birth

Table 2 shows hazard ratios for breast cancer according to size at birth. The ratios tended to rise with increasing birthweight,

**Table 1** Maternal, neonatal and childhood characteristics of 3447 women born at Helsinki University Central Hospital during 1924–33

| | Mean (SD) | Range | No. of missing values |
|---|---|---|---|
| Maternal | | | |
| Height (m) | 1.58 (0.06) | 1.04–1.86 | 245 |
| Weight in late pregnancy (kg) | 66.8 (8.7) | 45–134 | 261 |
| Body mass index in late pregnancy (kg/m$^2$) | 26.7 (3.1) | 19.4–51.1 | 285 |
| Age (years) | 27.7 (5.8) | 15–48 | 9 |
| Primiparous (%) | 42.0 | | 3 |
| Neonate | | | |
| Birthweight (g) | 3315 (493) | 1470–5600 | 0 |
| Head circumference (cm) | 34.3 (1.4) | 28.0–39.0 | 10 |
| Birth length (cm) | 49.7 (2.0) | 38.0–59.0 | 12 |
| Ponderal index (kg/m$^3$) | 26.9 (2.4) | 15.6–50.0 | 12 |
| Placental weight (g) | 628 (125) | 200–1290 | 7 |
| Length of gestation (days) | 276 (15) | 197–307 | 179 |
| % born before 37 weeks gestation | 10.1 | | 179 |
| Child | | | |
| Height (m) at age 7 years | 1.19 (0.05) | 0.98–1.37 | 0 |
| at age 11 years | 1.39 (0.06) | 1.13–1.61 | 0 |
| at age 15 years | 1.58 (0.06) | 1.29–1.81 | 0 |
| Weight (kg) at age 7 years | 21.7 (2.9) | 14.1–37.9 | 0 |
| at age 11 years | 32.1 (5.1) | 18.2–64.5 | 0 |
| at age 15 years | 49.7 (7.7) | 26.2–92.9 | 0 |
| Body mass index (kg/m$^2$) at age 7 years | 15.3 (1.3) | 11.7–24.1 | 0 |
| at age 11 years | 16.5 (1.7) | 12.2–31.2 | 0 |
| at age 15 years | 19.8 (2.5) | 12.3–41.2 | 0 |
| No. of people in house | 4.5 (1.5) | 2–14 | 570 |
| No. of rooms in house | 1.7 (0.8) | 1–7 | 536 |
| Crowding (no. of people/no. of rooms)* | 2.8 (1.6) | 0.6–11 | 576 |

*Log transformed.

*© 2001 Cancer Research Campaign*

**Table 2** Hazard ratios for breast cancer according to size at birth

| Variable | No. of cases | No. of women | Hazard ratio (95% CI) |
|---|---|---|---|
| Birthweight (g) | | | |
| ≤2500 | 6 | 191 | 1.0 |
| 3000 | 31 | 708 | 1.4 (0.6–3.4) |
| 3500 | 84 | 1420 | 1.9 (0.8–4.3) |
| 4000 | 41 | 880 | 1.5 (0.6–3.5) |
| >4000 | 15 | 248 | 1.9 (0.7–5.0) |
| Hazard ratio per kg = 1.22 (0.90 to 1.65) *P* = 0.2 | | | |
| Birth length (cms) | | | |
| <48 | 27 | 726 | 1.0 |
| 49 | 30 | 595 | 1.4 (0.8–2.3) |
| 50 | 65 | 1077 | 1.6 (1.0–2.5) |
| 51 | 32 | 588 | 1.5 (0.9–2.5) |
| >51 | 23 | 449 | 1.4 (0.8–2.4) |
| Hazard ratio per cm = 1.06 (0.98 to 1.15) *P* = 0.13 | | | |

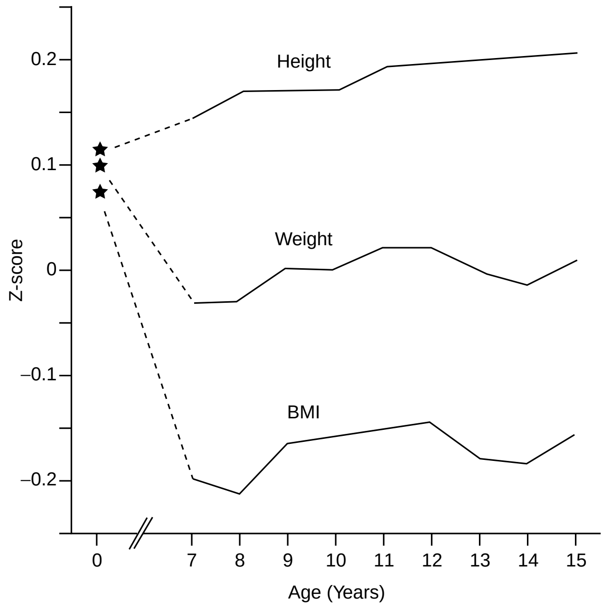

**Figure 1** Height, weight and body mass index during childhood, expressed as standard deviation (Z) scores, in women who later developed breast cancer although this was not statistically significant. The trend with birth length was similar, though also not significant. Hazard ratios were not related to placental weight nor to the length of gestation. The trends in Table 2 were little changed by adjusting for gestation.

### Growth in childhood

Figure 1, based on the Z scores, shows that at each age from 7 to 15 years the women who developed breast cancer were, on average, taller than the other women ($P < 0.05$ at every age). At 7 years, for example, their height was 0.8 cm above the average, while at 15 years it was 1.3 cm above. In contrast, women who developed breast cancer were thinner than the other women at all ages from 7 to 15 years ($P < 0.05$ at each age). Their body mass index at 7 years was 0.3 kg/m$^2$ below the average and at 15 years it was 0.4 kg/m$^2$ below the average. The changes in Z-score for height, weight and body mass index from 7 to 15 years were not statistically significantly different from those of the other women.

Table 3 shows hazard ratios for breast cancer according to height and body mass index at 7 and 15 years. The trends for increased breast cancer risk with increasing height and falling body mass index were both statistically significant. Body mass index at 7 years was not strongly correlated with birthweight (correlation coefficient = 0.15). In a simultaneous analysis, the hazard ratio for breast cancer was 1.21 (95% CI 1.06–1.38, $P$ = 0.004) for every kg/m$^2$ decrease in body mass index at 7 years and 1.27 (95% CI 0.97–1.78, $P$ = 0.08) for every kilogram increase in birthweight. There was no interaction between birthweight and body mass index in their effect on breast cancer risk.

### Maternal characteristics

As expected, taller mothers tended to have taller daughters, the correlation coefficient between mothers' and daughters' height at 7 being 0.36; and mothers with higher body mass indices had daughters with higher body mass indices, correlation coefficient being 0.23 at age 7 years. However, mothers' heights, weights and body mass indices during pregnancy were unrelated to the occurrence of breast cancer in their daughters. Similarly, the mothers' ages and parities were not related to their daughters' breast cancer risk.

### DISCUSSION

The present study investigated associations between early growth and breast cancer risk in a cohort of 3447 Finnish women, born in Helsinki, most of whom developed the disease after the age of 50 years, that is post-menopausally. We found that high birth weight and long body length at birth were associated with increased risk of the disease, although the association did not reach statistical significance. These borderline associations are similar to those found in a cohort of Swedish women born in Uppsala (Ekbom et al, 1992).

In addition to size at birth, we were able to examine how growth between 7 and 15 years modified the risk of breast cancer. We found that women who developed the disease had above average height through childhood but were relatively thin, with a below average body mass index. We did not have information on adult body mass index, and it is possible that these associations with childhood height and body mass are mediated through effects of adult body mass on breast cancer risk. However, it is clear that childhood body mass did not mediate the effects of high

 *© 2001 Cancer Research Campaign*

**Table 3** Hazard ratios for breast cancer according to body size at 7 and 15 years

| Variable | No. of cases | No. of women | Hazard ratio (95% CI) |
|---|---|---|---|
| Height at age 7 (cm) | | | |
| ≤114.5 | 22 | 641 | 1.0 |
| 117.5 | 32 | 703 | 1.3 (0.8–2.3) |
| 120 | 39 | 694 | 1.7 (1.0–2.8) |
| 123 | 41 | 722 | 1.7 (1.0–2.9) |
| >123 | 43 | 687 | 1.9 (1.1–3.1) |
| *P* for trend = 0.01 | | | |
| Height at age 15 (cm) | | | |
| ≤153 | 23 | 657 | 1.0 |
| 157 | 34 | 750 | 1.3 (0.8–2.2) |
| 160 | 33 | 720 | 1.3 (0.8–2.2) |
| 163 | 38 | 604 | 1.8 (1.1–3.1) |
| >163 | 49 | 716 | 1.9 (1.2–3.2) |
| *P* for trend = 0.005 | | | |
| BMI at age 7 (kg/m$^2$) | | | |
| ≤14.3 | 46 | 701 | 1.9 (1.2–3.1) |
| 14.9 | 34 | 682 | 1.4 (0.8–2.4) |
| 15.5 | 45 | 715 | 1.8 (1.1–2.9) |
| 16.2 | 27 | 642 | 1.2 (0.7–2.1) |
| >16.2 | 25 | 707 | 1.0 |
| *P* for trend = 0.009 | | | |
| BMI at age 15 (kg/m$^2$) | | | |
| ≤18 | 49 | 769 | 1.7 (1.0–2.7) |
| 19 | 33 | 596 | 1.4 (0.9–2.4) |
| 20 | 38 | 649 | 1.5 (0.9–2.4) |
| 21.5 | 29 | 724 | 1.0 (0.6–1.7) |
| >21.5 | 28 | 709 | 1.0 |
| *P* for trend = 0.03 | | | |

birthweight, since they had independent and opposite effects on breast cancer risk.

Earlier studies have shown that, after the menopause, tall women are more prone to develop breast cancer than shorter women (Cold et al, 1996; Vatten and Kvinnsland, 1990; Ziegler, 1997). Our data show that the association of tallness with breast cancer may reflect a greater height from birth onwards. Increased breast cancer risk was associated with increased length at birth followed by above average height at every age from 7 to 15 years. Previously, the Nurses' Health Study (Berkey et al, 1999) found an association between high peak height growth velocity, the most growth attained during any single year of adolescence, and an increased risk of breast cancer. The observation, however, was based on estimates of growth velocity derived from recalled body fatness at 10 years, menarcheal age and adult height. The results of our study, which are based on actual measurements of height during childhood, show that the velocity of height growth does not predict breast cancer risk.

Girls who are tall during childhood tend to have an earlier menarche than short girls (Ellison, 1981; Marshall and De Limongi, 1976) and our observations that tall girls exhibit increased breast cancer risk may partly explain the association between breast cancer and early menarche (Hulka and Stark, 1995). Greater height in childhood is associated with higher plasma concentrations of insulin-like growth factor-1 (IGF-1) (Juul and Skakkebaek, 1997; Nilsson et al, 1994), and this growth factor plays an important role in determining onset of puberty (Hiney et al, 1996; Juul et al, 1995). Furthermore, high plasma IGF-1 concentrations in pre-menopausal women are associated with increased breast cancer risk (Hankinson et al, 1998). IGF-1 is therefore a possible link between childhood height, age at menarche and breast cancer.

Puberty occurs when a girl reaches a critical weight/body mass, and overweight girls tend to experience menarche earlier than girls with normal body weight (Frisch and McArthur, 1974). It might be expected that overweight girls would have increased breast cancer risk. However, neither earlier prospective data (Le Marchand et al, 1988), nor retrospective data (Berkey et al, 1999; Magnusson et al, 1998) support this. Two retrospective case-control studies were based on information about childhood body mass as it was recalled during adult life before or after breast cancer diagnosis (Berkey et al, 1999; Magnusson et al, 1998). It is important to note that few girls in Finland were obese 60 years ago, when women in our cohort were children. The highest BMI category at age 15 was > 21.5 kg/m$^2$, which according to present standards represents normal body size. We cannot therefore conclude that childhood obesity, as defined in clinical practice today, protects against breast cancer.

Recent animal studies may provide clues to the biological mechanisms that link high childhood body mass index to a low risk of breast cancer. In animals exposed to oestrogens in early life, the mammary glands may differentiate early and are less susceptible to developing tumours upon exposure to carcinogens (Grubbs et al, 1985; Nagasawa et al, 1974). In humans, body mass index is an indicator of oestrogenicity, especially before puberty when adipose tissue is the major site of oestrogen release. Perhaps being overweight in childhood induces early breast differentiation and eliminates some undifferentiated mammary epithelial cells as targets for malignant transformation. Another possibility is that the oestrogenic effects of high childhood body mass increase the

*© 2001 Cancer Research Campaign*

expression of tumour suppressor genes, such as *BRCA1* (Hilakivi-Clarke 2000). This gene is known to be over-expressed during puberty (Marquis et al, 1995). It is induced by oestrogens (Gudas et al, 1995; Spillman and Bowcock, 1996) and its activity is associated with increased breast differentiation (Rajan et al, 1996), maintenance of the genomic integrity and DNA repair (Gowen, 1998).

In conclusion, we have found that women who developed breast cancer were tall and thin at all ages from 7 to 15 years. We speculate that these associations reflect the effects of high plasma concentrations of insulin-like growth factors, and a protective effect of high body mass through early breast differentiation.

### ACKNOWLEDGEMENTS

We thank Drs Marc Lippman and Bruce Trock, and graduate student Kerrie Bouker from Lombardi Cancer Center, Georgetown University for their constructive comments and critical reading of the manuscript. We thank Terttu Nopanen, Tiina Saarinen, Hillevi Öfverström-Anttila, Liisa Toivanen, Arja Purtonen, Tiina Valle, Hanna Pehkonen and Ulla Tarvainen for abstracting the data from the records. Sigrid Rosten was responsible for data management.

**Funding**: Cancer Research Foundation of America, Susan G, Komen Breast Cancer Foundation, and US Department of Defense (LH-C), and British Heart Foundation, Jahnsson Foundation, Finska Läkaresällskapet, Orion Corporation Research Foundation, and Finnish Foundation for Cardiovascular Research.

### REFERENCES

Berkey CS, Frazier AL, Gardner JD and Colditz G (1999) Adolescence and breast carcinoma risk. *Cancer* **85**: 2400–2409

Braun MM, Ahlbom A, Floderus B, Brinton LA and Hoover RN (1995) Effect of twinship on incidence of cancer of the testis, breast, and other sites (Sweden). *Cancer Causes Control* **6**: 519–524

Cerhan JR, Kushi LH, Olson JE, Rich SS, Zheng W, Folsom AR and Sellers TA (2000) Twinship and risk of postmenopausal breast cancer. *J Nat Cancer Inst* **92**: 261–265

Cleary ML and Maihle NJ (1997) The role of body mass index in the relative risk of developing premenopausal breast cancer. *Proc Soc Exp Biol Med* **216**: 28–43

Cold S, Hansen S, Overvad K and Rose C (1998) A woman's build and the risk of breast cancer. *Eur J Cancer* **34**: 1163–1174

Colditz GA and Frazier AL (1995) Models of breast cancer show that risk is set by events of early life: prevention efforts must shift focus. *Cancer Epidemiol Biomarkers Prev* **4**: 567–571

deWaard F and Trichopoulos D (1988) A unifying concept of the aetiology of breast cancer. *Int J Cancer* **41**: 666–669

Ekbom A, Trichopoulos D, Adami HO, Hsieh CC and Lan SJ (1992) Evidence of prenatal influences on breast cancer risk. *Lancet* **340**: 1015–1018

Ellison PT (1981) Prediction of age at menarche from annual height increments. *Am J Phys Anthropol* **56**: 71–75

Forsen T, Eriksson JG, Tuomilehto J, Teramo K, Osmond C and Barker DJP (1997) Mother's weight in pregnancy and coronary heart disease in a cohort of Finnish men: follow up study. *BMJ* **315**: 837–840

Forsen T, Eriksson JG, Tuomilehto J, Osmond C and Barker DJP (1999) Growth in utero and during childhood among women who developed coronary heart disease. *BMJ* **319**: 1403–1407

Frisch RE and McArthur JW (1974) Menstrual cycles: fatness as a determinant of minimum weight for height necessary for their maintenance or onset. *Science* **185**: 949–951

Gerhard I, Vollmar B, Runnebaum B, Klinga K, Haller U and Kubli F (1987) Weight percentile at birth: II prediction by endocrinological and sonographic measurements. *Eur J Obstet Gynecol Reprod Biol* **26**: 313–328

Gowen LC, Avrutskaya AV, Latour AM, Koller BH and Leadon SA (1998) BRCA1 required for transcription-coupled repair of oxidative DNA damage. *Science* **281**: 1009–1012

Grubbs CJ, Farneli DR, Hill DL and McDonough KC (1985) Chemoprevention of n-nitro-n-methylurea-induced mammary cancers by pretreatment with 17beta-estradiol and progesterone. *J Natl Cancer Inst* **74**: 927–931

Gudas JM, Nguyen H, Li T and Cowan KH (1995) Hormone-dependent regulation of BRCA1 in human breast cancer cells. *Cancer Res* **55**: 4561–4565

Hankinson SE, Willett WC, Colditz GA, Hunter DJ, Michaud DS, Deroo B and Rosner B, Speizer FE and Pollak M(1998) Circulating concentrations of insulin-like growth factor-I and risk of breast cancer. *Lancet* **351**: 1393–1396

Hilakivi-Clarke L, Clarke R, Onojafe I, Raygada M, Cho E and Lippman ME (1997) A maternal diet high in n-6 polyunsaturated fats alters mammary gland development, puberty onset, and breast cancer risk among female rat offspring. *Proc Natl Acad Sci USA* **94**: 9372–9377

Hilakivi-Clarke L (2000) *BRCA1*, estrogens and breast cancer. *Cancer Res* **60**: 1–10

Hiney JK, Srivastava V, Nyberg CL, Ojeda SR and Dees WL (1996) Insulin-like growth factor I of peripheral origin acts centrally to accelerate the initiation of female puberty. *Endocrinology* **137**: 3717–3728

Huang Z, Hankinson SE, Colditz GA, Stampfer MJ, Hunter DJ, Manson JE, Hennekens CH, Rosner B, Speizer FE and Willett WC(1997) Dual effects of weight and weight gain on breast cancer risk. *JAMA* **278**: 1407–1411

Hulka BS and Stark AT (1995) Breast cancer: cause and prevention. *Lancet* **346**: 883–887

Hunter DJ and Willett WC (1996) Nutrition and breast cancer. *Cancer Causes Control* **7**: 56–68

Johnson MR, Abbas A and Nicolaides KH (1994) Maternal plasma levels of human chorionic gonadotrophin, oestradiol and progesterone in multifetal pregnancies before and after fetal reduction. *J Endocrinol* **143**: 309–312

Juul A, Dalgaard P, Blum WP, Bang P, Hall K, Michaelsen KF, Muller J and Skakkebaek NE (1995) Serum levels of insulin-like growth factor (IGF) -binding protein -3 (IGFBP-3) in healthy infants, children, and adolescents: the relation in IGF-i, IGF-II, IGFBP-1, IGFBP-2, age, sex, body mass index, and pubertal maturation. *J Clin Endocrinal Metab* **80**: 2534–2542

Juul A and Skakkebaek NE (1997) Prediction of the outcome of growth hormone provocative testing in short children my measurement of serum levels of insulin-like growth factor I and insulin-like growth binding protein 3. *J Pediatr* **130**: 197–204

Le Marchand L, Kolonen LN, Early ME and Mi MP (1988) Body size at different periods of life and breast cancer risk. *Am J Epidemiol* **128**: 137–152

Magnusson C, Baron J, Persson I, Wolk A, Bergstrom R, Trichopoulos D and Adami HO (1988) Body size in different periods of life and breast cancer risk in post-menopausal women. *Int J Cancer* **76**: 29–34

Marquis ST, Rajan JV, Wynshaw-Boris A, Xu J, Yin GY, Abel KJ, Weber BC and Chodosh LA (1995) The developmental pattern of BRCA1 expression implies a role in differentiation of the breast and other tissues. *Nat Genet* **11**: 17–26

Marshall WA and De Limongi Y (1976) Skeletal maturity and the prediction of age and menarche. *Ann Hum Biol* **3**: 235–243

Michels KB, Trichopoulos D, Robins JM, Rosner BA, Manson JE, Hunter D, Colditz GA, Hankinson SE, Speizer FE and Willett WC (1996) Birthweight as a risk factor for breast cancer. *Lancet* **348**: 1542–6

Nagasawa H, Yanai R, Shonodo M, Nakamura T and Tanabe Y (1974) Effect of neonatally administered estrogen and prolactin on normal and neoplastic mammary growth and serum estradiol-17 level in rats. *Cancer Res* **34**: 2643–2646

Nilsson A, Ohlsson C, Isaksson OG, Lindahl A and Isgaard J (1994) Hormonal regulation of longitudinal bone growth. *Eur J Clin Nutr* **48**: S150–S158

Potischman N, Swanson CA, Siiteri P and Hoover RN (1996) Reversal of relation between body mass and endogenous estrogen concentrations with menopausal status. *J Natl Cancer Inst* **88**: 756–758

Rajan JV, Wang M, Marquis ST and Chodosh LA (1996) Brca2 is coordinately regulated with Brca1 during proliferation and differentiation in the mammary epithelial cells. *Proc Natl Acad Sci USA* **93**: 13078–13083

Royston P (1991) Constructing time-specific reference ranges. *Stat Med* **10**: 675–690

Sanderson M, Williams M, Malone KE, Stanford JL, Emanuel I, White E and Daling JR (1996) Perinatal factors and risk of breast cancer. *Epidemiology* **7**: 34–37

Spillman M and Bowcock A (1996) BRCA1 and BRCA2 mRNA levels are coordinately elevated in human breast cancer cells in response to estrogen. *Oncogene* **13**: 1639–1645

Trentham-Dietz A, Newcomb PA, Storer BE, Longnecker MP, Baron J, Greenberg ER and Willett WC (1997) Body size and risk of breast cancer. *Am J Epidemiol* **145**: 1011–1019

Trichopoulos D (1990) Hypothesis: does breast cancer originate in utero? *Lancet* **355**: 939–940

Vatten LJ and Kvinnsland S (1990) Body height and risk of breast cancer. A prospective study of 23,831 Norwegian women. *Br J Cancer* **61**: 881–885

Ziegler RG (1997) Anthropometry and breast cancer. *J Nutr* **127**: 924S–928S

Yong LC, Brown CC, Schatzkin A and Schairer C (1996) Prospective study of relative weight and risk of breast cancer: the Breast Cancer Detection Demonstration Project follow-up study, 1979 to 1987–1989. *Am J Epidemiol* **143**: 985–995

 *© 2001 Cancer Research Campaign*
